# Outbreak of febrile illness caused by coxsackievirus A4 in a nursery school in Beijing, China

**DOI:** 10.1186/s12985-015-0325-1

**Published:** 2015-06-18

**Authors:** Jin-Song Li, Xiao-Gen Dong, Meng Qin, Zhi-Ping Xie, Han-Chun Gao, Jun-Yong Yang, Xiao-Xin Yang, Dan-Di Li, Jie Li, Zhao-Jun Duan

**Affiliations:** National Institute for Viral Disease Control and Prevention, China CDC, 100 Ying-Xin St., Xuan-Wu District, Beijing 100052 China; Fengtai District Center for Disease Control and Prevention of Beijing, 3 Xi An St., Feng-Tai District, Beijing 100071 China

**Keywords:** Coxsackievirus A4, Outbreak, Preschool, Prevention, Control

## Abstract

**Background:**

Coxsackievirus A4 (CV-A4) is classified as human *enterovirus* A according to its serotype. CV-A4, an etiological agent of hand, foot, and mouth disease, affects children worldwide and can circulate in closed environments such as schools and hospitals for long periods.

**Findings:**

An outbreak of febrile illness at a nursery school in Beijing, China, was confirmed to be caused by CV-A4. Phylogenetic analysis of the complete genome of the isolated strain showed that the virus belongs to the same cluster as the predominant CV-A4 strain in China. This outbreak was controlled by effective measures.

**Conclusions:**

The early identification of the pathogen and timely intervention may be the most critical factors in controlling an outbreak caused by CV-A4 in a preschool.

## Findings

Human *enteroviruses* (HEVs) are a group of positive-sense single-stranded RNA viruses belonging to the genus *Enterovirus*, in the family *Picornaviridae*. HEVs are associated with hand, foot, and mouth disease (HFMD), herpangina and even death, and cause both sporadic infections and outbreaks, which often peak in summer and early autumn. Herpangina, predominantly caused by coxsackieviruses, often manifests as high fever, sore throat, and reduced appetite.

The genus *Enterovirus* includes 12 species. CV-A4 is classified as HEV-A based on its serotype, and is an etiological agent of HFMD [1–3]. The virus can be detected in throat swabs from herpangina patients [4], and can cause severe central nervous system symptoms [5]. CV-A4 can circulate in closed environments for long periods, which may be attributable to its prolonged shedding in stools, poor hand hygiene, and/or close contact [6]. CV-A4 has also been isolated from drinking water [7] and river water [8].

Here, we identified a febrile illness outbreak associated with CV-A4 in a preschool in China. The school, located in an urban district of Beijing, comprised six classes with 25–30 children in each class. One hundred sixty-five children, aged < 7 years, were in the care of 40 staff members. The class with the most cases of illness had 27 members, whereas another class with fewer cases had 26 children. “Case” is defined as any individual with symptoms that included a fever, cough, runny nose, and pharyngeal inflammation or a sore throat. “Intimate contact” is defined as any staff member or student with none of these symptoms. “Transmission rate” and “transmission period” are defined as reported previously [6]. The demographic and clinical data for the study subjects were obtained from the school and informed consent was given by the parents/guardians of the subjects.

On 24 May 2011, a 4 year-old boy presenting with a mild fever (38.1 °C) and pharyngeal inflammation was diagnosed with an acute upper-respiratory infection. The number of cases then increased rapidly. By 10 June 2011, 23 children in the preschool showed similar symptoms: six were clinically confirmed as suffering an upper-respiratory tract infection and 17 had herpangina. Twenty cases were from Class One and the other three were from a nearby class. Their ages ranged from 3 to 5 years (mean age, 4.3 years) and the sex ratio (male:female) was 18:5 (3.6:1). Most of the children displayed a self-limiting illness, lasting for 2–4 days. All the patients had a good prognosis and there were no fatalities during this outbreak.

**Fig. 1:**
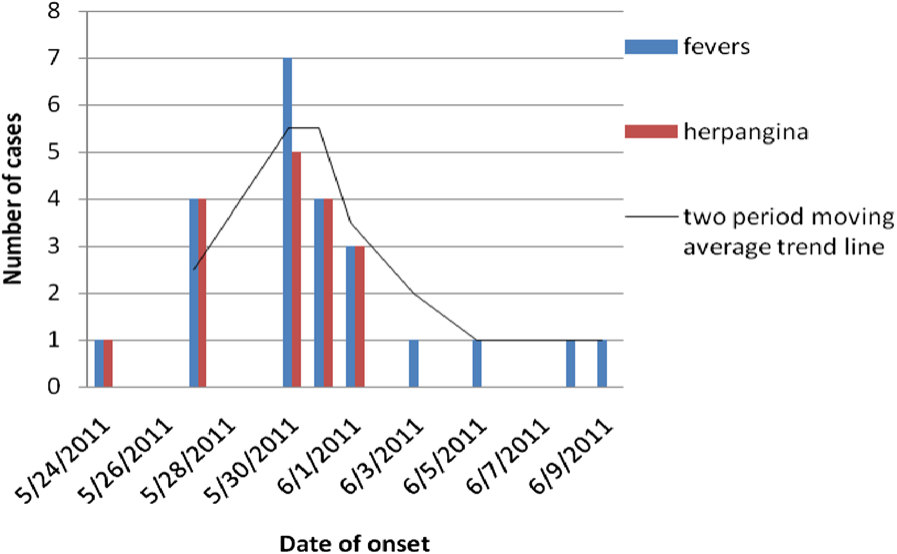
Epidemiological data for the febrile illness outbreak caused by CV-A4. Graph showing the number of cases and their clinical symptoms. The trend line for the average number of cases indicates that the outbreak began on 24 May, peaked on 30 May (after intervention), and ended on 10 June

The most common symptoms in the affected children were fever (100 %; range, 37.9–41.0 °C) and pharyngeal inflammation or sore throat (21/23, 91.3 %). One boy had a cough and three had runny noses. No other symptoms were noted in any of the subjects. The mean duration of fever was 2.70 days (95 % confidence interval, 2.34–3.05). In seven cases, the peak body temperature was > 38.9 °C. Leukocytosis (white blood cell count > 15 × 10^9^/L) was noted in four cases (Table 1). The peak incidence was between 27 May and 1 June, accounting for 78.3 % (18/23) of cases (Fig. 1).

**Table 1:** The symptoms observed for each of the cases during the outbreak and the pathogens isolated

No.	Sample name	Date of sample	Temperature (°C)	Cough	Pharyngeal inflammation or sore throat	Nasal discharge	WBC	Virus isolation	CPE
1	case1	6/1/2011	39	N	Y	N	WBC 15 × 10^9^/L	NE	UN
2	case2	6/1/2011	38.6	N	Y	N	UN	CV-A4	P
3	case3	6/1/2011	38.5	N	Y	N	UN	CV-A4	NE
4	case4	6/1/2011	38.1	N	Y	N	UN	CV-A4	NE
5	case5	6/1/2011	41	N	Y	N	UN	CV-A4	P
6	case6	6/1/2011	39.4	N	Y	N	UN	CV-A4, HAdV-3	P
7	case7	6/1/2011	39.4	N	N	Y	WBC 12 × 10^9^/L	CV-A4	P
8	case8	6/1/2011	38.5	N	Y	N	WBC 12.6 × 10^9^/L	Influenza B	UN
9	case9	6/1/2011	38	N	Y	Y	UN	CV-A4	P
10	case10	6/1/2011	37.9	N	Y	N	UN	CV-A4	P
11	case11	6/1/2011	37.9	N	Y	Y	UN	CV-A4	P
12	case12	6/1/2011	39.8	N	Y	N	UN	CV-A4	P
13	case13	6/3/2011	39	Y	Y	N	WBC 17 × 10^9^/L	CV-A4	P
14	case14	6/3/2011	38	N	Y	N	UN	CV-A4	P
15	case15	6/3/2011	38.8	N	Y	N	WBC 15 × 10^9^/L	CV-A4	NE
16	case16	6/3/2011	39	N	Y	N	UN	CV-A4	P
17	case17	6/3/2011	39	N	Y	N	WBC 17.8 × 10^9^/L	NE(bacteria positive)	UN
21	Intimate contact1	6/8/2011	normal	/	/	/	UN	NE	UN
23	Intimate contact2	6/8/2011	normal	/	/	/	UN	NE	UN
24	Intimate contact3	6/8/2011	normal	/	/	/	UN	NE	UN
25	Intimate contact4	6/8/2011	normal	/	/	/	UN	NE	UN
26	Intimate contact5	6/8/2011	normal	/	/	/	UN	NE	UN
27	Intimate contact6	6/8/2011	normal	/	/	/	UN	HRV-C,HAdV-3,CV-A4	P
28	Intimate contact7	6/8/2011	normal	/	/	/	UN	NE	UN
29	case18	6/8/2011	38.1	N	Y	N	UN	HRV-A	NE
30	case19	6/8/2011	39.2	N	N	N	UN	CV-A4,HAdV-3	NE
31	case20	6/8/2011	38.1	N	Y	N	UN	CV-A4	NE
32	case21	6/8/2011	38	N	Y	N	UN	CV-A4	NE
33	Intimate contact8	6/8/2011	normal	/	/	/	UN	CV-A4,HRV-A	NE
34	Intimate contact9	6/8/2011	normal	/	/	/	UN	CV-A4,HRV-A	NE
35	Intimate contact10	6/8/2011	normal	/	/	/	UN	NE	UN
36	Intimate contact11	6/8/2011	normal	/	/	/	UN	CV-A4,HRV-A	NE
37	case22	no sample	38.3	N	Y	N	UN	UN	UN
38	case23	no sample	37.9	N	Y	N	UN	UN	UN

*N*, no symptoms; *Y*, symptoms observed; /, none of the symptoms observed; *UN*, undetected; *NE*, no viruses isolated from samples or cells; *P*, CV-A4 was isolated from cells; *WBC*, white blood cell; *CV-A4*, coxsackievirus A4; *HAdV-3*, human adenovirus 3; *HRV-A*, human rhinovirus A; *CPE*, cytopathic effect

Thirty-two oropharyngeal swabs were taken from 21 patients (no samples were available for two cases) and 11 intimate contacts from the two classes. All 32 swab specimens were stored in virus preservation solution at −70 °C until analysis. The nucleic acids were extracted with a QIAamp MinElute Virus Spin Kit (Qiagen, Valencia, CA, USA), according to the manufacturer’s instructions. All the samples were screened by PCR or RT–PCR for influenza A and B viruses, respiratory syncytial virus, human rhinovirus, human echovirus, parainfluenza viruses 1, 2, 3 and 4, human metapneumovirus, human coronavirus NL63, human adenovirus, and some HEVs, as reported previously [9–13]. The positive PCR products were purified and sequenced.

Of the 32 oropharyngeal swabs collected, 21 were positive for CV-A4. The detection rates in the patients and their intimate contacts were 81.0 % (17/21) and 36.4 % (4/11), respectively. Human adenovirus-3, human rhinovirus, and influenza B were also detected, but the samples were negative for the other viruses tested (Table 1). Outbreaks of human coxsackievirus infections in preschools are reported frequently worldwide and are typically associated with HFMD caused by the coxsackie A16 virus [14]. However, febrile outbreaks caused by CV-A4 are rarely reported in preschools. In this study, 17 of the 21 tested cases were CV-A4 positive, strongly suggesting that this outbreak was caused by CV-A4.

Herpangina diseases are asymptomatic or self-limiting infections, often caused by EV71 or coxsackieviruses, including CV-A4. Chia-Jie Lee reported that herpangina caused by CV-A4 usually presented with high fever > 39.0 °C (75.5 %), oral ulcers (80.6 %), and leukocytosis (34.0 %), and that fever lasted longer than 3 days in 23.5 % of sufferers [15]. In another outbreak in a preschool class, the transmission rate was 25.9 % (7/27) and the transmission period was 28 days [6]. In our study, 42.9 % (9/21) of cases had high fever, and the fever persisted for more than 3 days in 14.3 % of patients (3/21) and for 3 days in 42.9 % of patients (9/21). The transmission rate was 74.1 % (20/27) in Class One, the transmission period was 18 days, and two CV-A4-positive patients had leukocytosis (leukocyte counts ≥ 15,000 /μL). We did not measure the white blood cell counts in all cases.

The virus was isolated from all CV-A4-positive throat samples in human rhabdomyosarcoma cells, using a previously reported protocol [16]. Cytopathic effects were observed in 52.2 % (12/21) of the samples incubated with rhabdomyosarcoma cells. Twelve VP1 sequences and three complete genomes of CV-A4 (two from initial cases and one from intimate contact FT27) were amplified from the cell culture supernatants by RT–PCR and rapid amplification of cDNA ends–PCR, according to the manual supplied with the SMARTer™ RACE cDNA Amplification Kit (Japan, Tokyo) [17]. The primer sequences and details of the reaction conditions are given in Table 2. The genome of CV-A4 is about 7460 bp and the VP1-encoding fragment is 915 bp. The genomes of three strains (FT27, FT7, and FT5) were 98.5–99.5 % homologous and the VP1 fragments of nucleic acids were 99.9–100 % homologous. These sequences shared 95.5–96.5 % identity with the clinical CV-A4 strain previously isolated in mainland China (GenBank: HQ728260). As expected, the polyproteins of FT7 and the CV-A4 mainland strain (GenBank: HQ728260) shared 96.5 % identity (VP4, 97.1 %; VP2, 96.7 %; VP3, 96.5 %; and VP1, 96.2 %) and the nonstructural proteins shared 95.8 % identity (2A, 95.1 %; 2B, 98.3 %; 2C, 95.3 %; 3A, 94.0 %; 3B, 98.5 %; 3C, 96.7 %; and 3D, 96.2 %).

**Table 2:** Primers used to amplify the complete CV-A4 genome

Primer name	Primer sequence	Fragment (bp)
F1	TTTAAAACAGCCTGTGGGTTG	~300
R1	AACCCATAGGCAGGCCGCC	
cox241	ACCCGGCTAACTACTTCGAGAAAC	1853
cox2093	ATGAATGAGCCCGTAAACATGAAA	
M1908	AGGTGTAAGCCGGTTGCTCATAC	1025
M2932	CACTGGAACGATTCTCGAGCATC	
VP1A(2319)	CTTCGTAGTGCCACCAGACAC	1066
VP1S(3384)	AGCTCCAGATTGTTGACCGA	
N2846	TCACCTTCGTCACCAATCTAG	1012
N3857	CCTGGAGACTGCGTCAGTGA	
bcox3615	CAGTGAGTACTACCCTGCCAGGTATCAA	1318
bcox4932	TGCGGTGTTATTTTCAGAGCACAGTTTG	
ccox4610	AGCAAGTGGTCACTGTCATGGATGA	1324
ccox5933	CCTGCACAAAAGCCCTGCCTGCCAT	
dcox5856	TGGAGGAGTAGTTACATCAGTTGGA	1548
dcox7403	CAGTTATGTTCACGACCAGATTTCT	
ecox-a(race pcr 3′)	ATGCCAATGAAGGAGATTCATGAGTCC	~300

**Fig. 2:**
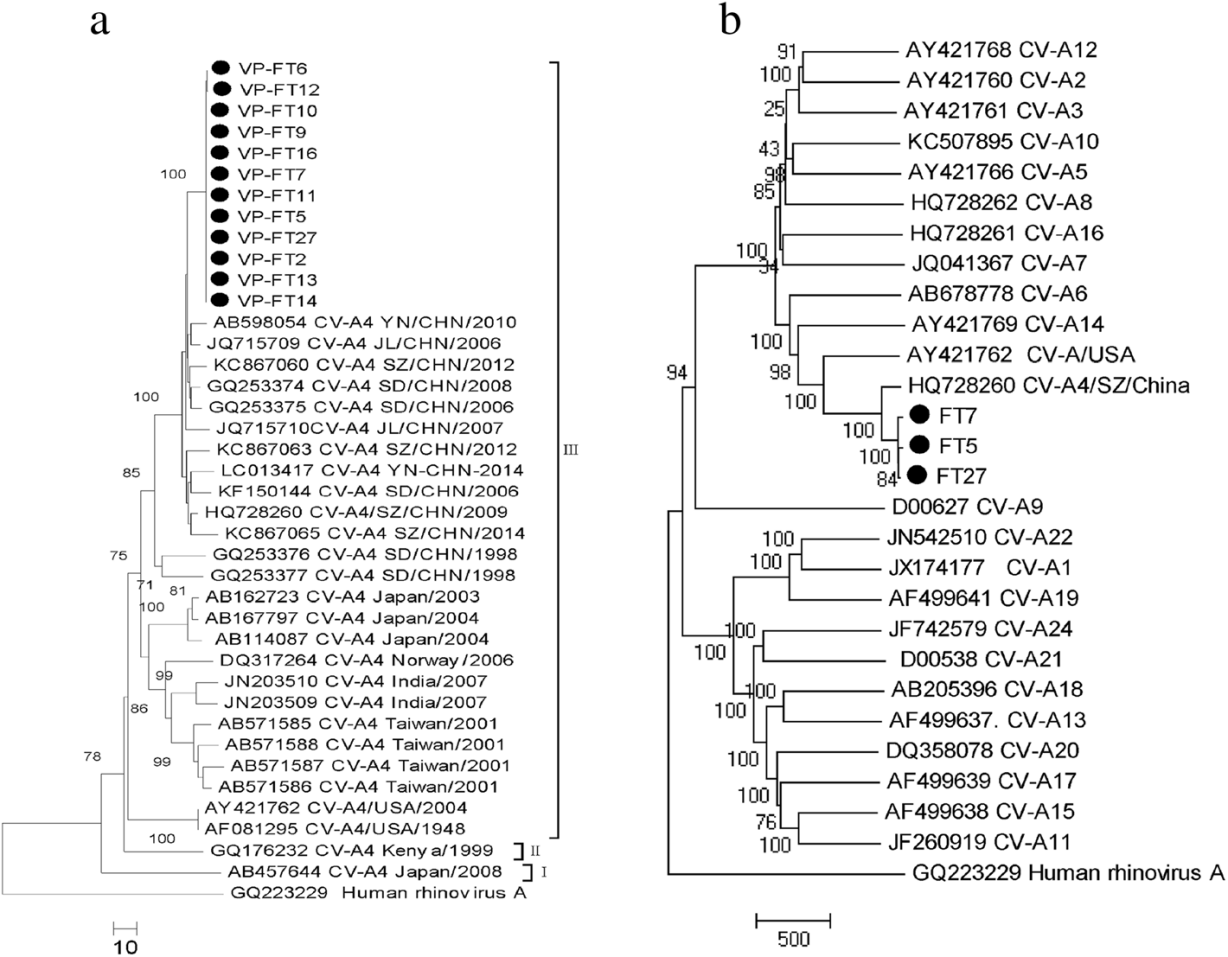
Phylogenetic analysis of the pathogens isolated during the outbreak. A phylogenetic analysis of the nucleotide sequences of the viruses isolated during the outbreak: (**a**) the three partial VP1 sequences of CVA-4; (**b**) the near-complete nucleotide sequences of strains of human Coxsackie A virus. Phylogenetic analysis was performed and the tree was constructed using the neighbor-joining algorithm implemented in the MEGA version 5.0 software with 1000 bootstrap pseudoreplicates. The numbers on the branches indicate the bootstrap values, excluding those of < 70 % for clarity. Human rhinoviruses (HRVs) were used as outgroups on both trees. Red dots show the CV-A4 strains isolated in this study and the black dots show the only complete genome of CV-A4 in the GenBank database

In a phylogenetic analysis of the near-complete genome of the CV-A4 strain isolated in this outbreak, it clustered with the only two complete genomes of CV-A4 deposited in the GenBank database (Fig. 2). Analysis of a 3′ partial VP1 sequence, showed all CV-A4 isolates from this outbreak clustered on the same branch and were closely related to the predominant CV-A4 strains JL/CHN/2006 (GenBank: JQ715709) and SD/CHN/2008 (GenBank: GQ253374). All CV-A4 isolates from mainland China, Japan, India, and Taiwan, including CV-A4-mainland (GenBank: HQ728260), formed a subgenogroup, and isolates from the USA formed another subgenogroup within genotype III. Global CV-A4 strains show regional variations and were classed into three genotypes based on their 3′ partial VP1 sequences [3, 18]. Our results indicate that the CV-A4 strain isolated in this study probably also varies across its geographic distribution (Fig. 2). This CV-A4 strain is the predominant strain in China, resulting from the rapid lineage turnover of CV-A4 and the replacement of previously circulating strains by this new dominant variant [18].

CV-A4 is primarily transmitted via the fecal–oral route by contaminated hands, toys, and food. When the local Center for Disease Control received the report of this outbreak on June 1, normal control measures were taken, such as hand-washing, disinfection of the classroom, and body temperature screening. Health education was intensified and the public activities at all of the nursery schools were cancelled, as described previously [19, 20]. On June 1, the children in Classes One and Two celebrated Children’s Day with a party. On June 3, when the pathogens causing this outbreak were identified, the local Center for Disease Control suspended Class One for 10 days. During the outbreak, 20 children in Class One and three children in Class Two caught the disease. Although the transmission period of CV-A4 within a class is reported to be 28 days (6), this outbreak was controlled within 18 days. All the control measures used in this outbreak were similar to those used for HMFD caused by EV71.

## Conclusions

This outbreak of febrile illness at a preschool in Beijing, China, was caused by CV-A4. Measures, such as surveillance in peak season, early identification of the pathogen and timely intervention, are key to controlling outbreaks of disease caused by CV-A4 or other HEVs.

### Nucleotide sequence accession numbers

The three complete genomes have been submitted to the GenBank database under accession numbers KP676984, KP676985, and KP676986. Partial sequences of CV-A4 and other viral sequences have been deposited under accession numbers KP676948–KP676983.

## Abbreviations

CV-A4: Coxsackievirus A4
HEV: Human enterovirus
HFMD: Hand, foot, and mouth disease
